# Expert elicitation on ultrafine particles: likelihood of health effects and causal pathways

**DOI:** 10.1186/1743-8977-6-19

**Published:** 2009-07-24

**Authors:** Anne B Knol, Jeroen J de Hartog, Hanna Boogaard, Pauline Slottje, Jeroen P van der Sluijs, Erik Lebret, Flemming R Cassee, J Arjan Wardekker, Jon G Ayres, Paul J Borm, Bert Brunekreef, Kenneth Donaldson, Francesco Forastiere, Stephen T Holgate, Wolfgang G Kreyling, Benoit Nemery, Juha Pekkanen, Vicky Stone, H-Erich Wichmann, Gerard Hoek

**Affiliations:** 1 Dutch National Institute for Public Health and the Environment (RIVM), Bilthoven, the Netherlands; 2 University of Utrecht, Institute for Risk Assessment Sciences, Utrecht, The Netherlands; 3 University of Utrecht, Copernicus Institute, Utrecht, the Netherlands; 4 Institute of Occupational & Environmental Medicine, University of Birmingham, Birmingham, UK; 5 Centre of Expertise in Life Sciences, Hogeschool Zuyd, Heerlen, The Netherlands; 6 Julius Center for Health Sciences and Primary Care, University Medical Center Utrecht, Utrecht, The Netherlands; 7 University of Edinburgh Centre for Inflammation Research, Edinburgh, UK; 8 Department of Epidemiology, Health Authority Rome, Italy; 9 University of Southampton, School of Medicine, UK; 10 Institute of Lung Biology and Focus Network Nanoparticles and Health, Helmholtz Center Munich, Neuherberg/Munich, Germany; 11 Lung Toxicology Research Unit, K.U. Leuven, Leuven, Belgium; 12 National Public Health Institute, Unit of Environmental Epidemiology, Kuopio, Finland; 13 School of Life Sciences, Napier University, Edinburgh, UK

## Abstract

**Background:**

Exposure to fine ambient particulate matter (PM) has consistently been associated with increased morbidity and mortality. The relationship between exposure to ultrafine particles (UFP) and health effects is less firmly established. If UFP cause health effects independently from coarser fractions, this could affect health impact assessment of air pollution, which would possibly lead to alternative policy options to be considered to reduce the disease burden of PM. Therefore, we organized an expert elicitation workshop to assess the evidence for a causal relationship between exposure to UFP and health endpoints.

**Methods:**

An expert elicitation on the health effects of ambient ultrafine particle exposure was carried out, focusing on: 1) the likelihood of causal relationships with key health endpoints, and 2) the likelihood of potential causal pathways for cardiac events. Based on a systematic peer-nomination procedure, fourteen European experts (epidemiologists, toxicologists and clinicians) were selected, of whom twelve attended. They were provided with a briefing book containing key literature. After a group discussion, individual expert judgments in the form of ratings of the likelihood of causal relationships and pathways were obtained using a confidence scheme adapted from the one used by the Intergovernmental Panel on Climate Change.

**Results:**

The likelihood of an independent causal relationship between increased short-term UFP exposure and increased all-cause mortality, hospital admissions for cardiovascular and respiratory diseases, aggravation of asthma symptoms and lung function decrements was rated medium to high by most experts. The likelihood for long-term UFP exposure to be causally related to all cause mortality, cardiovascular and respiratory morbidity and lung cancer was rated slightly lower, mostly medium. The experts rated the likelihood of each of the six identified possible causal pathways separately. Out of these six, the highest likelihood was rated for the pathway involving respiratory inflammation and subsequent thrombotic effects.

**Conclusion:**

The overall medium to high likelihood rating of causality of health effects of UFP exposure and the high likelihood rating of at least one of the proposed causal mechanisms explaining associations between UFP and cardiac events, stresses the importance of considering UFP in future health impact assessments of (transport-related) air pollution, and the need for further research on UFP exposure and health effects.

## Background

Epidemiological studies have in recent decades convincingly shown that exposure to ambient particulate air pollution is associated with increased cardiovascular and respiratory morbidity and mortality [1-3] as well as cancer [1,4,5]. Particulate matter (PM) is a complex mixture, and as yet much remains unknown about the agents within air pollutant mixtures that cause the adverse health effects. Recently, outcomes of toxicological and epidemiological studies suggest that the ultrafine component of PM might play a role in initiating or stimulating part of these health effects [6]. Ultrafine particles (UFP; i.e. atmospheric particles with aerodynamic diameter of < 100 nm) make up only 10% of the total mass of PM_2.5_, but in number UFP dominate the PM mixture as they constitute up to 90% of the aerosol. Therefore, UFP have a large number concentration as well as a relatively large surface to volume ratio. Ambient UFP are mainly combustion-derived being produced by the transport and industry sectors.

Currently, health effects related to UFP exposure have been studied less extensively than effects of fine and coarse PM. The mechanisms at cell and molecular level are as yet only partly understood [1,2,6-14]. There are multiple competing causal models and hypotheses. This implies that uncertainty is not just related to what parameters to use in impact estimations, but more fundamentally, about the assumed causal mechanisms themselves [15]. One of the first hypotheses regarding the specific working mechanism of UFP was posed by Seaton and co-workers [16]. They suggested that a systemic inflammatory response to particulate air pollution could be caused by the large surface area of UFP. This might cause or aggravate cardiovascular and other diseases. Since then, several studies and reviews have been published about the potential mechanisms relating UFP to (cardiovascular) health effects [1,2,4,6-8,10,11,13,14,17-19]. These studies have elaborated on the hypothesis suggested by Seaton and have also provided new theories about plausible pathways, including translocation of particles to the bloodstream and subsequently to other organs [1,4,8,11], and particles altering respiratory reflexes and acting on the autonomic nervous system (ANS) [1]. All these processes may eventually lead to a cardiac event, either by stimulating plaque rupture which can subsequently lead to thrombosis [20]; or by affecting the ANS potentially leading to arrhythmia. The suggested causal mechanisms vary (substantially) between authors and it is not always evident how plausible a specific hypothesized pathway is. However, it should be recognised that these different pathways are not mutually exclusive. In addition, some (parts) of these pathways may also underlie health effects related to coarser PM fractions (e.g. airway inflammation), whereas others are thought to be specific to UFP, such as translocation of particles into the blood or other organs.

Significant health gains could be achieved by implementing policy measures to reduce air pollution. Health Impact Assessments (HIA) are often carried out in order to develop or evaluate such policies. These assessments need information about causal relationships and concentration-response functions. However, for UFP no accepted summary concentration-response function is available. Moreover, there are hardly any long-term exposure studies [6,7,12]. Therefore, these HIAs have often considered PM_2.5 _or PM_10 _effects for which such knowledge is available. The potential health effects of UFP have been largely ignored in HIA. Omission of UFP in HIA may lead to an under-estimation of the health impacts of e.g. policies designed to reduce transport-related air pollution, because reductions of UFP concentrations may be more substantial in response to these policies than reductions in fine PM concentrations.

In order to judge the possibility to include UFP in HIA, first an assessment of the likelihood of a causal relationship between UFP exposure and relevant health endpoints (e.g. cardiac events such as myocardial infarction) independent from effects of coarser fractions of other components of air pollution is required. Evidence for pathophysiological mechanisms explaining these events add to the biological plausibility of epidemiologically observed associations and may give clues as to which components of the mixture are important in causing adverse health effects. Evidence on mechanisms can subsequently help in formulating effective counter measures. One way to address these problems in the absence of sufficient published studies is to use expert judgment approaches to assess the degrees of (dis)agreement between different experts. We used an expert elicitation workshop to assess the likelihood of causality, selected causal pathways and concentration-response functions for UFP.

### Expert elicitation

Expert elicitation is a systematic approach to generate and synthesize subjective judgments of relevant experts on a subject where well-established knowledge has not yet been developed due to uncertainty stemming form e.g. insufficient, contradicting, low-quality or unattainable data. The process seeks to make the (un)published knowledge and wisdom of experts explicit and utilizable, based on their accumulated experience and expertise, including their insight into the limitations, strengths and weaknesses of the published knowledge and available data. A formal expert elicitation in 2007 [21] quantified concentration-response functions and uncertainty for PM_2.5_-related mortality. Other (less formal) expert workshops have for example focused on potential mechanisms and methods for testing hypotheses on cardiovascular effects of air pollution [13], and reviewed the evidence for cardiovascular and neurodegenerative effects of ambient particles [4].

The primary aim of our two day expert elicitation was to bring together epidemiologists, toxicologists and clinicians that are renowned for their expertise in the field of health effects of UFP to examine the plausibility of causal relationships existing between UFP exposure and health effects. The secondary aim was to quantify concentration-response functions. We chose a multidisciplinary approach to allow for potentially different opinions in the environmental health field and to promote synthesis of knowledge and discussion. This paper describes the results of the first day of the workshop, dealing with causality issues. The quantification of specified concentration-response functions is described in a separate paper (Hoek *et al*., submitted).

## Methods

### Expert selection

Key European experts on UFP and health from clinical, toxicological and epidemiological backgrounds were selected in a two-step systematic peer-nomination. As a first step, we approached nominators, inviting: (a) first, second and last authors that had published at least two papers within the field of UFP and health in peer-reviewed journals (key words of literature search: ultrafine particles or particle number concentration or PM0.1 or UFP *and *epidemiology or health or effects or toxicology), as well as (b) scientists that participated in the WHO systematic review of air pollution and coordinators of leading EU projects in this research field (based on information at ). These nominators were asked to nominate 5 toxicologists, 5 epidemiologists and 5 clinicians who had, in their opinion, the educational background and/or experience to display both a thorough understanding of results from the epidemiological and toxicological literature addressing the relationship between UFP and various health effects, and to be able to evaluate these results in the context of other evidence pertinent to air pollution and various health effects. The nominators were allowed to nominate themselves. For budgetary reasons, the nominated experts had to be based in Europe.

As a second step, we invited the top 5 ranked scientists within each of the three mentioned disciplines, based on the responses from the 43 identified nominators. Only one expert per research group was invited to attend. Invited experts who did not agree or were unable to participate were replaced with the next candidate from the list within their discipline, provided that they were nominated by at least 5 nominators. The following experts (per discipline in which they were nominated and in alphabetical order) accepted and attended the workshop. Toxicologists: Prof. Dr. P. Borm, Prof. Dr. K. Donaldson, Prof. Dr. W. G. Kreyling, Prof. Dr. V. Stone. Epidemiologists: Prof. Dr. B. Brunekreef, Dr. F. Forastiere, Prof. Dr. J. Pekkanen, Prof. Dr. E. Wichmann. Clinicians: Prof. Dr. J. Ayres, Prof. Dr. S. Holgate, Prof. Dr. B. Nemery, Prof. Dr. A. Seaton. Two other experts (one epidemiologist and one toxicologist) also accepted, but were eventually unable to attend.

The workshop was prepared by a team of the University of Utrecht (Institute for Risk Assessment Sciences and Copernicus Institute) and the Dutch National Institute for Public Health and the Environment (RIVM) and consisted of Anne Knol, Jeroen de Hartog, Hanna Boogaard, Pauline Slottje, Jeroen van der Sluijs, Erik Lebret, Flemming Cassee and Gerard Hoek.

### Protocol, briefing book and workshop structure

In order to plan and structure the expert elicitation and produce transparent and traceable results, we developed a formal protocol based on protocols used for similar workshops [21,22]. This protocol outlined the structure of the expert elicitation sessions. In order to allow the experts to prepare for the meeting, we composed a briefing book with a reading guideline, references of 81 peer reviewed studies on UFP and health, summary texts and tables of the literature, preliminary graphical representations of the potential causal mechanisms, plus two recent papers on expert elicitation for PM_2.5 _[21,22]. This briefing book comprised of most papers that were selected in the literature review, which was carried out by the team of RIVM and University of Utrecht. Slightly less animal studies as compared to epidemiological studies were included, because some animal studies that were adequately summarized by the provided toxicological reviews were not included. Experts were specifically asked to check and complement the literature.

The causal pathway diagrams as presented to the experts have been added in the appendix. Both the protocol and briefing book were distributed to the panel beforehand on a pass-word protected temporary website, and were available to the experts during the meeting both on CD and as hard copies. The workshop started with an introduction by a member of the organizing team (JvdS) about the process of expert elicitations, the ratings scales and potential heuristic biases related to expert judgements. It was made clear to the experts that we did not necessarily aim at reaching consensus among them, but rather at gaining insight into the various prevailing opinions and underlying assumptions and uncertainties.

The approach taken during the workshop, to evaluate causal mechanisms relating UFP exposure to cardiac events, is similar to 'group model building' (GMB) methods [23,24] for joint exploration of causal models. Three types of tasks can be identified in GMB exercises [24,25]: (1) 'divergent tasks': elicitation of information on what to include in the model, e.g. gathering ideas on possible causes, effects, and pathways; (2) 'convergent tasks': combining the elicited information into a chart, model, collective problem description, or policy strategy; (3) 'evaluation phase': discussing and evaluating the model that has been developed, and prioritizing issues for further discussion or elaboration.

Workshop participants only performed task three. The other two stages were performed by the research team. Information about model components was collected through literature review and two overview models were created representing six different potential causal pathways. Participants evaluated these proposed pathways.

The experts were instructed to take account of the following basic assumptions and restrictions. The focus was on total UFP from atmospheric origin, without differentiating between different sources of UFP or different components within the mixture (e.g. diesel, carbon black, fly ash). Furthermore, we considered inhalation as the only intake route, since it is thought to be the major portal of entry of UFP into the human body [11]. Finally, we focused specifically on the potential of UFP to *cause *effects *separately *and *independently *from effects of coarser fractions or other air pollution components. The morning session of the meeting dealt with the likelihood of a causal relationship between UFP exposure and various health endpoints. The health endpoints were selected *a priori *based on their relevancy for HIA, and therefore primarily associated with health endpoints as studied in epidemiology. During the session, the experts were given the opportunity to reflect on this *a priori *list and discuss adding or dismissing specific health endpoints. In the afternoon session, the likelihoods of six *a priori *selected causal mechanisms associating UFP with cardiac events were examined. We did not aim to discuss the specific details of particular (patho)physiological processes or working mechanisms within a particular pathway. Instead, we focused on the likelihood of these six broadly defined pathways, based upon previous reviews.

For clarity and to enhance inter-expert comparability, the formulation of some questions was slightly refined during the workshop, based on experts' suggestions. The final questions for the morning session were formulated as follows: *"Considering the evidence, how do you rate the likelihood that short-term/long-term exposure to ultrafine particles at realistic ambient levels is causally related to health endpoint X?" *The questions on plausible causal mechanisms were asked for cardiac events only, conditional on the existence of a causal relationship, and independent of each other (i.e. allowing for the possibility of singular as well as multiple routes in parallel). These questions were posed as follows: *"Assuming a causal relationship between exposure to ultrafine particles at realistic ambient levels and cardiac events, and considering the evidence, how do you rate the likelihood that these events can be (partly) explained by pathway Y?"*. A low rating could be given if any of the steps in the pathway was considered to be of little plausibility or importance.

**Figure 1:**
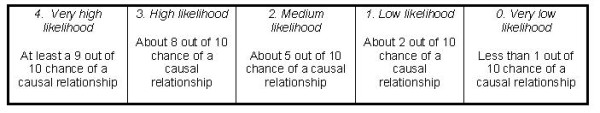
**Level of confidence scheme used for likelihood rating, adapted from **[27].

Proposed mechanisms were evaluated using a so-called 'Delphi' technique [26]. The experts were asked to rate the likelihoods of the various pathways using a number (0 – 4) according to a confidence scheme adapted from the one used by *inter alia *the Intergovernmental Panel on Climate Change (IPCC) [27] (figure 1). For each question, the following steps were performed: (1) an individual rating, (2) an (anonymous) on-screen graphical presentation of these initial ratings, (3) a plenary discussion based on this presentation, and (4) a final individual rating. We thereby encouraged experts to first reflect upon their own ideas, before sharing their knowledge and potentially adjusting their ratings based on new insights. We favoured group discussion over, for example, personal interviews, in order to encourage inter-expert communication in a multidisciplinary setting.

The questions related to causal pathways were preceded by a plenary discussion and some refinement of the graphical representations of these pathways. Experts were explicitly asked to provide both written and oral motivation for their initial and final ratings, e.g. on which evidence and assumptions these were primarily based. The results of the final ratings and a synopsis of the motivation and major points of discussion are described in this paper.

## Results

**Figure 2:**
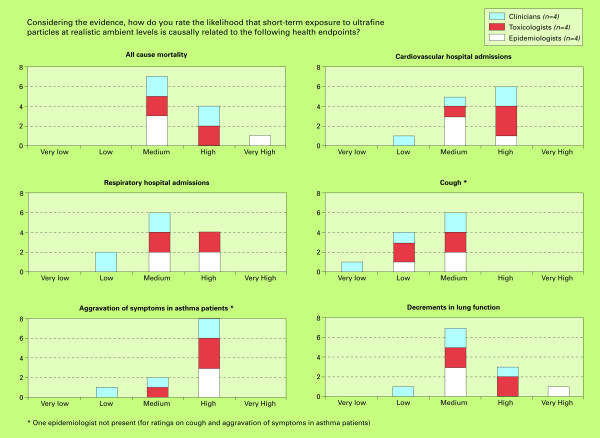
**Final likelihood ratings for health endpoints being causally related to short-term UFP exposure**.

**Figure 3:**
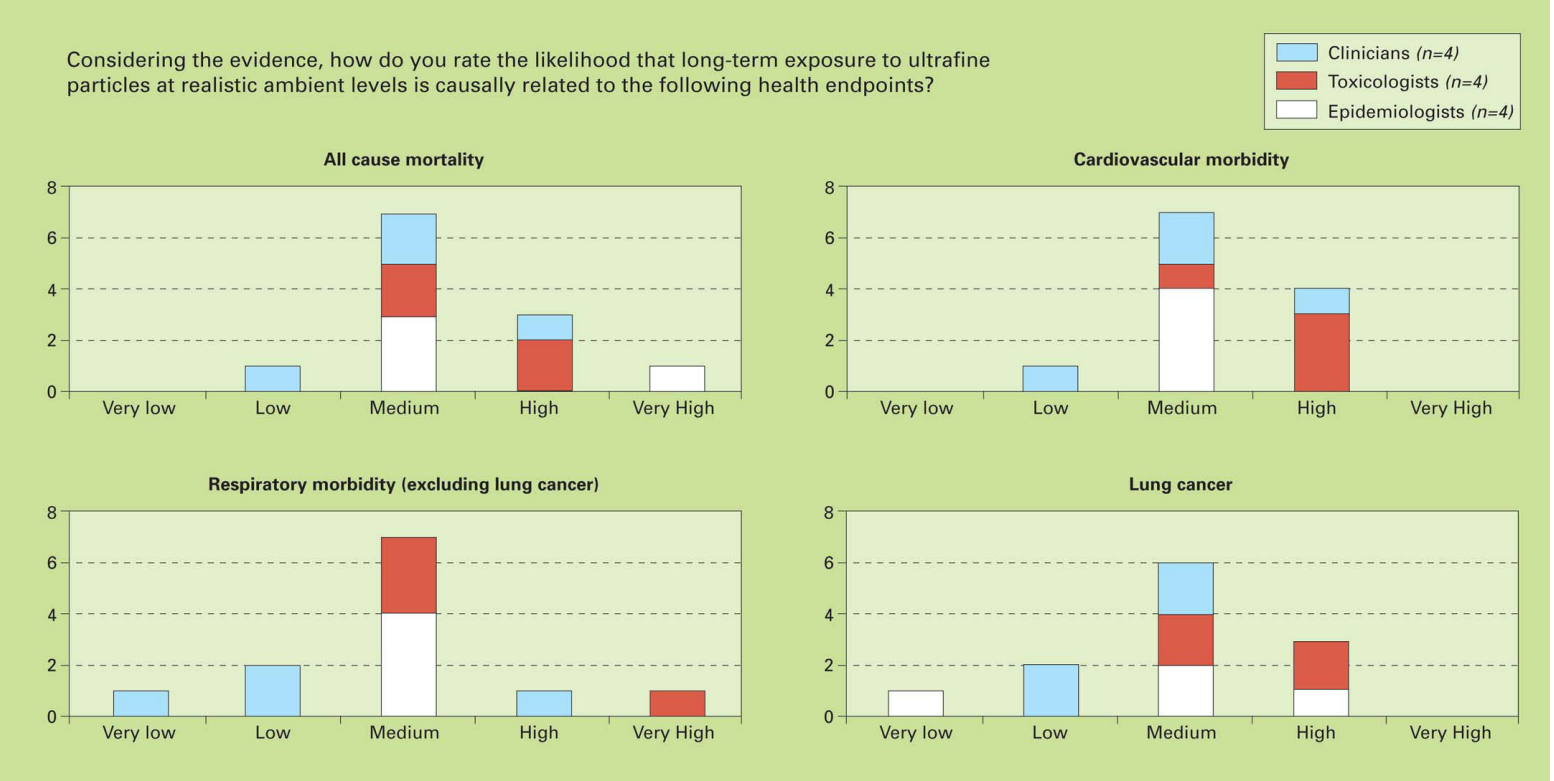
**Final likelihood ratings for health endpoints being causally related to long-term UFP exposure**.

**Figure 4:**
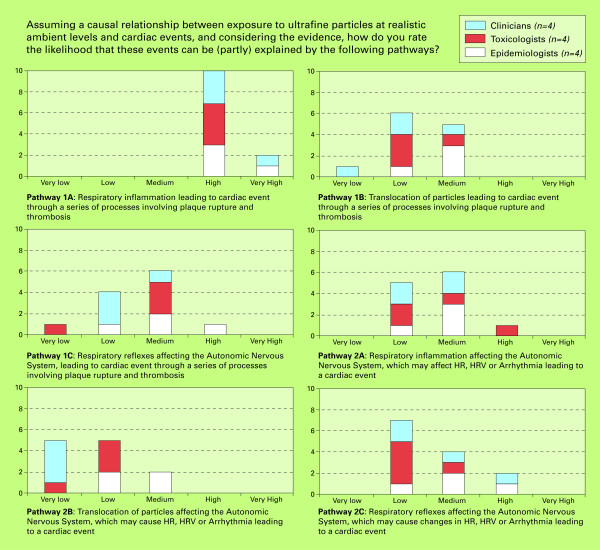
**Final likelihood ratings for causal pathways relating UFP exposure to acute cardiac events**.

The results of this study are divided into three sections: 1) the likelihood of a causal relationship between short-term UFP exposure and selected health outcomes; 2) the likelihood of a causal relationship between long-term UFP exposure and selected health outcomes; 3) the likelihood of selected potential causal mechanisms for UFP exposure and acute cardiac events. Figures 2, 3 and 4 show the numbers of experts providing specific likelihood ratings, which are expressed as categories ranging from very low to very high.

### 1. Likelihood of causal relationships between short-term UFP exposure and health effects

The results of the final expert ratings considering the likelihood that short-term UFP exposure is causally related to selected health endpoints are presented in figure 2.

#### All-cause mortality

Ratings of the likelihood of causality ranged from medium (n = 7) to very high. The experts giving higher ratings argued that underlying causal mechanisms for cardiovascular effects are very plausible and documented in toxicological studies, and that independent and stable effects of UFP have been found in epidemiological studies (quoted references included [28-30]). The ratings were based on the notion that short-term variation in *all-cause *mortality is primarily driven by variation in *cardiovascular *mortality. Evidence of health effects from other components such as NO_2 _and CO that are correlated with UFP raised their confidence. Recent unpublished work from London further raised confidence among some experts. Lower ratings were motivated by the fact that most studies rely on limited, rather incoherent and mainly indirect data from e.g. PM_2.5 _studies. Additionally, exposure misclassification, lack of evidence for independent effects of UFP and lack of correction for publication bias were mentioned by some experts. Data from Rome and Aberdeen that were unpublished at the time of rating were also mentioned to support lower ratings (data from Aberdeen are currently published in [31], a manuscript about the data from Rome is submitted (Belleudi *et al*., submitted)).

#### Cardiovascular and respiratory hospital admissions

The likelihood of a causal relationship between short-term UFP exposure and cardiovascular or respiratory hospital admissions was rated from low to high, with overall slightly higher ratings for cardiovascular hospital admissions (figure 2). For the latter, some experts considered several studies [32-34] to have clearly shown mechanistic relationships, with good evidence from epidemiological and human controlled exposure studies. The mechanisms for cardiovascular effects were generally considered very plausible. Lower ratings were mainly motivated by the limited number of studies, with mixed and controversial results. Independent effects of UFP were considered difficult to separate from other components of air pollution.

For respiratory hospital admissions, similar motivations were given. Most experts considered respiratory effects to be mechanistically plausible, although some argued UFP effects to be less convincing than effects for larger fractions (e.g. PM_2.5_) [35,36].

#### Respiratory symptoms

The respiratory outcomes considered were cough, aggravation of symptoms in asthma patients, and decrements in lung function. Of these, aggravation of symptoms in asthma patients was generally rated highest and cough lowest (figure 2). For cough, likelihood ratings ranged from (very) low to medium (n = 6). Low ratings were partly given because cough was considered to be a very non-specific endpoint which functions as an indicator of a range of health endpoints of varying severity. Also, several experts assessed that there is little evidence available for an association between UFP and cough, and cough was considered more likely to be related to larger PM fractions (high dust exposure). The experts generally considered aggravation of symptoms in asthma patients exposed to UFP highly likely (likelihood ratings ranging from low to high (n = 8)), even though they judged the clinical evidence to be rather inconsistent. Mechanistically, aggravation of symptoms was believed to be very plausible due to the high susceptibility of asthma patients, in which UFP may further irritate already stimulated cells. A study showing increased medication use [37], mechanistic studies among children in Helsinki [38] and respiratory effects shown in a London study [39] were quoted as references to support high ratings. Decrements in lung function (likelihood ratings medium (n = 7) to very high) were, like cough, considered to be a rather non-specific endpoint. Some studies [39-42] were quoted that support an effect of UFP on lung function.

### 2. Likelihood of causal relationships between long-term UFP exposure and health effects

The results of the final expert ratings considering the likelihood of long-term UFP exposure to be causally related to various health endpoints are presented in figure 3.

#### All cause mortality

A causal association between long-term UFP exposure and all-cause mortality was generally considered of medium (n = 7 out of 12) likelihood by the experts (likelihood ratings ranging from low to very high). The ratings of these long-term exposure effects were overall only slightly lower than for the corresponding short-term exposure effects. Evidence was deemed mostly indirect, based on associations between other PM fractions or other exposure variables and mortality. Nevertheless, some experts indicated that mechanistic and clinical studies do suggest an independent effect. The experts hypothesised that insoluble UFP may accumulate and remain in the lungs or secondary target organs for over 6 months, potentially leading to cardiovascular effects. Several experts also mentioned epidemiological studies investigating effects of residing in proximity to major roads, which they considered to be driven (at least partly) by UFP [43-46]. They reasoned that the contrasts in UFP concentrations are higher close to roads than for PM_10 _and PM_2.5_. Some experts mentioned that the database of studies relative to "proximity to roads" was limited and somewhat inconsistent or that the contribution of UFP to potential effects was difficult to disentangle. Lower ratings were mostly motivated by a lack of data.

#### Cardiovascular morbidity and respiratory morbidity (excluding lung cancer)

Likelihood ratings ranged from low to high, mostly medium (n = 7) for cardiovascular morbidity. The experts considered the underlying mechanism to be plausible, implying that UFP can affect all the elements of the main triad leading to cardiovascular disease (endothelial dysfunction, thrombosis and plaque destabilization). As for mortality, the ratings of these long-term exposure effects on cardiovascular morbidity were only slightly lower than for the corresponding short-term exposure effects.

The ratings for respiratory morbidity were more variable across experts (ranging from very low to very high), though most provided a rating of medium (n = 7). Likelihood of effects on respiratory morbidity were rated slightly lower compared to cardiovascular morbidity, and much lower than aggravation of asthma (its related short-term effects counterpart). As a potential mechanism, the experts reasoned that respiratory conditions, such as asthma and COPD, are affected by inflammation, which in turn can be caused by UFP exposure. This was supported by evidence from proximity to roads studies, increasing the confidence of the experts. Furthermore, it was argued that if cigarette smoke contains a lot of UFP and can cause COPD or emphysema, then ambient UFP could play a similar role. However, as for mortality, much uncertainty remains because effects of UFP cannot easily be disentangled from those caused by larger particles.

#### Lung cancer

Ratings for the likelihood of lung cancer effects ranged from very low to high (mostly medium, n = 6 out of 12). On the one hand, some experts referred to findings of excessive lung cancer in humans exposed to fine particles [47] as well as to the potency of UFP to cause lung cancer in animals. On the other hand it was mentioned that there are no specific studies on UFP effects and some experts thought it more likely that larger particles are responsible.

### 3. Likelihood of different causal pathways leading to a cardiac event

**Figure 5:**
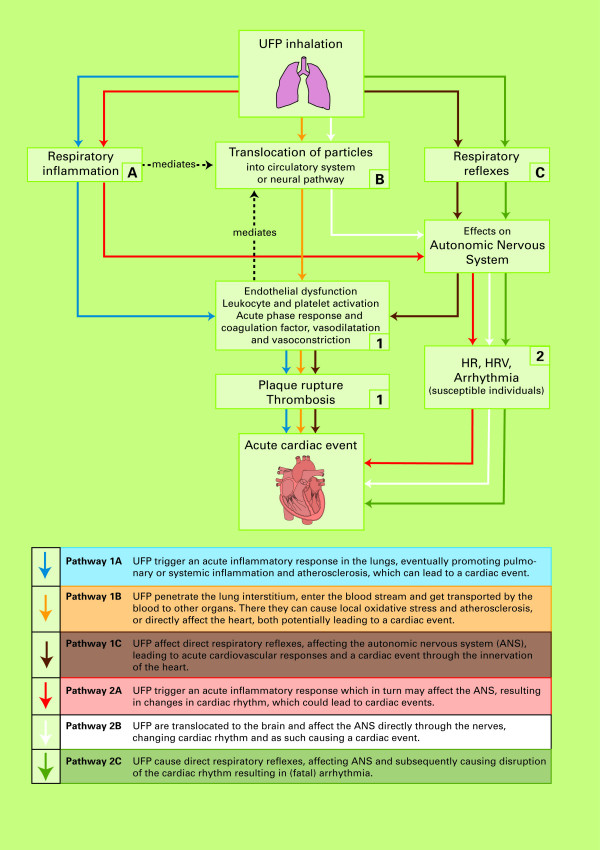
**Schematic overview of causal pathways potentially explaining the manifestation of acute cardiac events caused by UFP exposure**.

**Figure 6:**
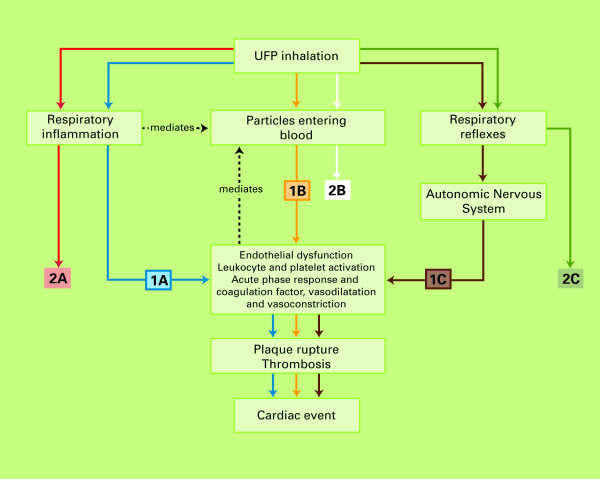
**Schematic overview of causal pathways potentially explaining the manifestation of acute cardiac events through thrombosis and plaque rupture, caused by UFP exposure**.

**Figure 7:**
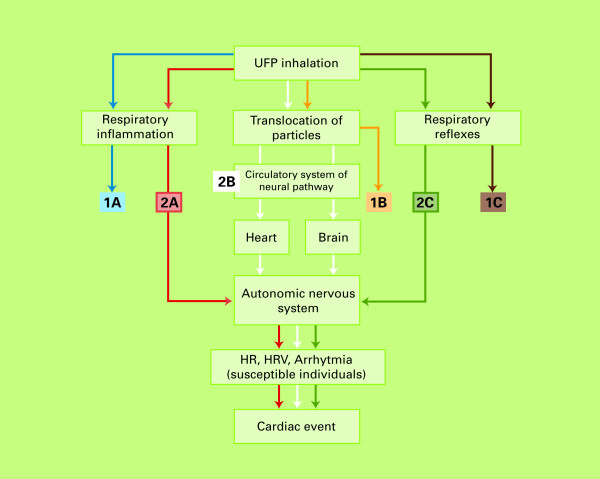
**Schematic overview of causal pathways potentially explaining the manifestation of acute cardiac events through effects on the autonomous nervous system, caused by UFP exposure**.

In total, six broadly defined causal pathways potentially explaining the role of UFP in contributing to cardiac events in humans were discussed and rated by the experts, represented together in figure 5. The elicitation was based upon two graphical displays of these mechanisms. Figures 6 and 7 in the appendix show the original drawings as presented to the experts during the meeting. These figures purposely provide a simplified representation of a highly complex reality. As a result, many important elements, such as the exact mechanisms of oxidative stress, transcription factors, or inflammatory mediators, could not be included in detail. The six pathways are further described in the appendix. The likelihood ratings of the experts for each of these pathways are presented below. In all of these ratings, a causal relationship between UFP and cardiac events was assumed. The ratings were thus conditional on causality.

#### Pathway 1a (airway inflammation – plaque rupture)

As shown in figure 4, pathway 1a was given the highest likelihood ratings by the experts: high (n = 10 out of 12) to very high. The experts thus had little doubt that this pathway explains at least part of the cardiovascular effect of UFP. Experts mainly motivated their ratings by referring to the large body of studies from different disciplines, which consistently point into the same direction. All steps within this causal pathway were considered to be proven, mainly in animal studies with more limited support from human data (e.g. from the AIRGENE project [48] and [49]).

#### Pathway 1b (translocation of particles – plaque rupture)

The experts rated the likelihood of this pathway from (very) low to medium, referring to the limited evidence that exists on the subject (mainly animal studies). Data also suggest that only a small proportion of UFP translocate. Most experts did believe that translocation occurs, but the dose might be too low to cause cardiac effects. Others argued that UFP might accumulate and cause effects, but these would probably take some years to develop (long-term effects).

#### Pathway 1c (respiratory reflexes – ANS – plaque rupture)

Figure 4 shows the rather broad range of ratings for pathway 1c (ranging from very low to high; with half of the experts rating medium), pointing at significant uncertainties and differences of opinion amongst experts. There exists a rather large body of evidence in animals for respiratory reflexes, but none in humans. In general, therefore, experts considered this pathway as uncertain and hence its likelihood as difficult to judge.

#### Pathway 2a (respiratory inflammation – ANS – arrhythmia)

The likelihood ratings for pathway 2a ranged from low to high, mostly medium (n = 6). Some experts doubted the importance of the ANS in relating UFP exposure to cardiac events as such, but noted that, if an effect on the ANS would occur, UFP induced inflammation would probably be its most likely cause. This explains why pathway 2a is rated slightly higher than 2b and 2c. Further evidence causing the experts to judge this pathway rather unlikely came from UFP studies in Edinburgh, in which healthy and diseased volunteers have been exposed to diluted diesel engine exhaust with and without UFPs (Mills *et al*., in preparation; [50]). These studies showed no effect on HRV despite a significant increase in cardiac events. However, some clinicians reasoned that this pathway is possible nonetheless, based on the plausibility of irritation of autonomic receptors by inflammatory mediators.

#### Pathway 2b (translocation of particles – ANS – arrhythmia)

The likelihood of pathway 2B was rated very low to medium by the experts, with 10 experts providing a low or very low rating. Especially clinicians were highly sceptical towards the likelihood of this pathway. Evidence was judged insufficient, and the amount and rate of translocation was not considered to be able to explain the onset and magnitude of the cardiac effects found.

#### Pathway 2c (respiratory reflexes – ANS – arrhythmia)

The likelihood of pathway 2c was rated low by most of the experts (n = 6), but also medium and high by some. Motivation for a low likelihood mainly involved the limited data available. However, some epidemiologists argued that several studies give proof for the different steps defining this pathway, and most clinicians found this mechanism to be plausible even if there is too little evidence to prove it.

## Discussion

A multidisciplinary European expert team rated the likelihood of causal relationships between ambient UFP exposure and selected health endpoints, including all-cause mortality and cardiovascular and respiratory hospital admissions. The experts also rated the likelihood of potential underlying causal mechanisms that may explain an effect of UFP on cardiac events. The likelihood of a causal relationship between short-term UFP exposure and all-cause mortality, cardiovascular and respiratory hospital admissions, aggravation of asthma symptoms and lung function decrements was rated as medium to high by most of the experts. The ratings for long-term exposure related effects were only slightly lower compared to short-term related effects for mortality and cardiovascular hospital admissions; long-term exposure effects on respiratory morbidity and lung cancer were mainly rated medium. Divergence of opinions among experts was larger for respiratory morbidity and lung cancer than for total mortality and cardiovascular morbidity. There does not appear to be much difference between the ratings of epidemiologists, toxicologists and clinicians, although our sample size is too small to make any definitive statements about potential interdisciplinary variation. Moreover, even though most experts were nominated in one specific category, they might in reality represent the views of multiple disciplines.

From the evaluated causal pathways relating UFP exposure to cardiac events, the pathway involving respiratory inflammation and subsequent thrombotic effects (pathway 1a) was rated most likely. All experts rated the likelihood of this pathway as high or very high. This pathway is most often described in the literature, and the specific steps of the causal pathway were all considered plausible. The lowest ratings were given to the pathway 2b describing translocation of particles affecting the autonomic nervous system (ANS), which in turn may lead to changes in HR, HRV or arrhythmia. For this specific pathway, evidence was considered missing or even contradictory, and the plausibility was thought to be limited. Other pathways involving effects on the ANS (1c, 2a and 2c) were given slightly higher ratings. However, overall, the experts expressed the route via plaque rupture/thrombosis to be more likely to cause a cardiac event than the route via the effects on the ANS. Although we confined the elicitation to mechanisms potentially explaining cardiac effects, (part of) the same or similar mechanisms may also explain the onset and progression of other adverse health effects. Similarly, parts of the proposed mechanisms might also play a role in explaining adverse health effects related to exposure to coarser fractions of PM, other components or air pollution or engineered nanomaterials [1,11,19,51].

The experts were explicitly asked to rate the likelihood of a causal relationship, given the available evidence (i.e. they were not asked to rate the availability of evidence). The general lack of consistent evidence made the process of rating a challenging task for most experts. Complicating factors included that information had to be extrapolated: from animal to humans; from larger particles (PM_2.5 _and PM_10_) to UFP; from high doses as frequently used in experimental studies to concentrations similar to those in ambient air [6]; from (time-series) monitoring measurements to personal exposure; and from the studied sample of the population to the general population. Additionally, experts indicated the uncertainty related to lag times and presence or absence of a threshold of effect, accuracy of death certification and hospital admissions, susceptible groups, as well as variation in the analytical methods employed [7]. The presence of these uncertainties supports the use of formal expert elicitation to assess the likelihood of causality and causal mechanisms.

The likelihood of a causal association between UFP and mortality was assessed as being lower than in a recent US expert panel assessing causality of PM_2.5 _and mortality [22]. In that study, 10 out of 12 experts rated the likelihood of causality as very high, one as high and one as medium (quantitative numbers translated into our schema using the nearest number). In our study, likelihood of a causal association between UFP and mortality was rated high or very high by 4 out of 12 experts, medium by 7 experts, and low by one. Besides being based on higher evaluations of the likelihood of a causal relation for PM_2.5 _as compared to UFP, the differences may also be partly due to the composition of the panel or other methodological differences between the studies. Furthermore, it is likely that the larger database of studies on long-term exposure of PM_2.5 _compared to UFP played an important role in the higher rating in the US study. There was no overlap between experts participating in the US study and those participating in our study.

The overall medium to high likelihood rating of causality of health effects of UFP exposure and the high likelihood rating of at least one plausible mechanism explaining associations between UFP and cardiac events, support the potential usefulness of inclusion of UFP in future Health Impact Assessments (HIA) involving air pollution. The elicited concentration-response functions that were derived on the second day of our expert meeting (Hoek *et al*. submitted) can be used as input for these assessments. HIAs usually assess the health effects of particulate matter air pollution based on concentration-response functions as derived for e.g. PM_2.5 _or PM_10_, [52] which as such function as a proxy for other (correlated) components of the air pollution mixture. However, PM_2.5 _and PM_10 _concentrations do not capture variations of UFP very well; hence the potential effects of UFP have been largely ignored. Especially in the assessment of transport-related air pollution this may be a limitation, as motorized transport affects UFP concentrations more than PM_2.5 _or PM_10_. Therefore, in order to provide improved estimates of the health effects of air pollution, UFP effects should be assessed separately. As such we have made a first attempt to fill an important hiatus in current HIA of air pollution. Compared to the evidence for PM_2.5_, there is still considerable uncertainty, calling for additional research. The results of this expert elicitation give a starting point for evaluating which aspects of the pathways could be focused on in further research.

We have only presented the final likelihood ratings, which were given after plenary discussion, while the individual initial ratings were given before any discussion on the particular question. In a post-hoc comparison of final and initial ratings, we saw that – although most experts stuck to their initial judgments – some did occasionally adjust their rating after group discussion. In those cases, the ratings were usually not changed more than one point (rating class) and mostly in the direction of the mean. We have no reason to believe this to be an effect of 'peer pressure'. Rather, adjustments to initial ratings were brought about by considering new arguments, as indicated in the written motivations, or by harmonization of interpretation of the question within the expert panel, which we think both have increased the value of the final ratings. We did not aim at reaching consensus among the experts. Given the limited state of knowledge in the field, the scientific debate should consider the full spectrum of reasonable hypotheses, and forcing conversion to a consensus view may lead to putting more weight to one of the hypotheses than warranted. Closure of the scientific debate should be based on empirical evidence and not in a negotiated consensus amongst expert judgments. Also, different estimates are often based on completely different motivations, and a central 'consensus' estimate might no longer relate clearly to anyone's viewpoints. Finally, we realize that we have been able to obtain a multidisciplinary sample of respected scientific opinions, but not a representative set of all opinions available on the subject matter in the scientific community. Possibly, the nomination procedure has biased the selection towards 'believers' of UFP as a causal agent. Nonetheless, the set of expert judgments we have assembled is expected to collectively enhance scientific understanding of the (likelihood of) health effects related to UFP exposure and helps to clarify the current state of knowledge.

## Conclusion

The overall medium to high likelihood rating of causality of health effects of ultrafine particle exposure and the high likelihood rating of at least one plausible causal mechanism explaining associations between ultrafine particles and cardiac events, supports the need to consider inclusion of ultrafine particles in future health impact assessments of (transport-related) air pollution.

## Abbreviations

ANS: Autonomic nervous system; CO: carbon mono-oxide; COPD: Chronic Obstructive Pulmonary Disease; GMB: Group model building; HIA: Health Impact Assessment; HR: Heart Rate; HRV: Heart Rate Variability; IPCC: Intergovernmental Panel on Climate Change; NO_2_:Nitrogen dioxide; PM: Particulate matter; RIVM: Dutch National Institute for Public Health and the Environment; UFP: Ultrafine particles; WHO: World Health Organisation.

## Competing interests

Prof. dr. Paul Borm is both co-author of this paper and Editor-in-Chief of this journal. He has had no involvement in the reviewing and publication process.

## Authors' contributions

AK, GH, JdH, HB, PS, JvdS and FC organized the workshop and prepared the material. PS, JvdS, AK and AW conducted research into methods for expert elicitation. JA, PB, BB, KD, FF, SH, WK, BN, JP, VS and EW attended the workshop as experts. AK prepared the main manuscript. All authors read and approved the final manuscript.

## Appendix: Potential causal mechanisms for UFP exposure and cardiac events

The initial (patho)physiological effects of UFP can broadly be divided into:

A. Respiratory and/or systemic inflammation;

B. Translocation of particles to the bloodstream; and

C. Respiratory reflexes and consequent dysfunction of the autonomic nervous system (ANS)

These three responses can all lead to a cardiac event, roughly caused by 2 different mechanisms:

1) Through a series of processes resulting in plaque rupture and thrombosis;

2) By affecting the ANS, which may lead to changes in HR, HRV or Arrhythmia.

Combining these potential sub-pathways, in total six broad (partly overlapping and all potentially co-existing) pathways can be identified (figures 6 and 7 – graphical versions as presented to the experts). These pathways are shortly described below.

### Lung deposition

All pathways considered here start with deposition of UFP, which enter the lungs by millions with every breath [4], on the respiratory epithelium. The smaller the particle, the higher the probability that it hits the epithelium of a lung structure [6]. The efficiency of deposition, along with the large surface area and potential of bound transition metals are thought to be very important for initiating a physiological response [11]. Many of the effects occurring straight after deposition, such as retention, clearance, dis-aggregation and distribution, are not yet fully understood [4,6,12], but the initial interaction between particles and the surfactant film is considered to set off a complex immunological cascade in the lung [4]. There is convincing evidence that UFP are able to retain in the epithelium and interstitium (the space in between cells) for prolonged periods of time [6], potentially leading to endothelial dysfunction (Mills *et al*. in preparation, [53]).

#### Pathway 1a

Pathway 1a describes the mechanism in which UFP trigger an acute inflammatory response in the lungs through oxidative stress via activation of oxidative stress-responsive transcription factors [12,19]. The inflammation can be mediated by transition metals (derived from fuel combustion) that are bound on the reactive surface of these particles, but there are potentially also non-transition metal-mediated pathways to inflammation, relating to the small size and large reactive surface of UFP. Cells such as macrophages, epithelial cells and neutrophilic granulocytes are subsequently activated and can produce reactive oxygen species (ROS), free radicals, hydrogen peroxide, etc, to attack the UFP. This process leads to secretion of cytokines and chemokines into the affected area. Subsequently, a cascade of events may trigger changes in the control of blood clotting and promote pulmonary or systemic inflammation and atherosclerosis. This can in turn lead to acute cardiovascular responses such as increased blood pressure, thrombosis, and eventually a cardiac event. This pathway, which was the one originally proposed by Seaton and co-workers [16], is generally considered important, based on both in vitro and in vivo studies [1].

#### Pathway 1b

In recent years, several studies have shown that a fraction of UFP can, unlike coarse or fine particles, penetrate deeply into the lung interstitium and evade clearance mechanisms [1]. As such, UFP may directly enter into the blood stream through phagocytosis by macrophages or endocytosis by the epithelial and endothelial cells. Subsequently UFP can translocate to extrapulmonary sites such as bone marrow, lymph nodes, liver, heart, spleen, and brain [1,4,8,11]. Evidence is however conflicting with regard to the extent of this translocation and its pathological impact [6,10], which is assumed to be dependent on particle size, chemical characteristics, and surface features [1]. The circulating particles may cause local oxidative stress that could destabilize atherosclerotic plaques and, similar to the mechanisms described for pathway 1a, set of a cascade of reactions involving plaque rupture, thrombosis and eventually acute cardiac events [1,11,54]. Oxidative stress can increase the permeability of the lung epithelium and thereby further increase potential for translocation of particles [12]. Alternatively, particles may cause a direct effect to the heart if transferred there by the bloodstream [11]. Furthermore, other molecules that are produced in the lung as a response to particles may also enter the interstitium and the bloodstream, potentially leading to various negative systemic effects [12].

#### Pathway 1c

A third hypothesis with regard to initiation of a physical response after exposure to UFP involves the suggested ability of UFP to stimulate nerve endings in the walls of the airways causing direct respiratory reflexes. Such stimuli may result in dysfunction of the autonomic nervous system (ANS) and cardiovascular autonomic dysfunction [1]. The most described effect that is assumed to be the result of this process is disruption of the cardiac rhythm resulting in (fatal) arrhythmia [1,7,11] (pathway 2c). However, effects on the ANS may also eventually lead to acute cardiovascular responses and a cardiac event [13,55,56] through the innervation of the heart. The latter mechanism is represented by pathway 1c.

#### Pathway 2a

Besides potentially resulting from direct stimulus of lung nerve ending, effects on the ANS can also be a response to respiratory inflammation as a result of cytokine release in an acute phase reaction [8]. This can potentially lead to changes in heart rate (HR), heart rate variability (HRV) or even arrhythmia, which suggests another pathway towards cardiac events [7,11]. Overall, evidence on the effects of particulate air pollution on blood pressure and HR remains inconsistent [8]. Effects are probably mainly or only possible in susceptible patients with pre-existing heart disease such as MI and chronic heart failure [7]. Increases in HR in response to air pollution are furthermore mostly found in people with high blood viscosity [12]. However, as yet much remains to be elucidated and further studies are needed to investigate UFP (or total PM) related ANS effects.

#### Pathway 2b

Some studies, as reviewed by Oberdörster [19], have investigated the potential of UFP to be translocated to the brain through neuronal uptake via transcytosis. The olfactory nerve is considered to be the most likely pathway for the transport of particles inhaled through the nose. However, it is not yet known whether or not these translocated UFP cause injury or toxicity to the brain [1,19]. In theory, they may affect the ANS, which may in turn affect cardiac rhythm and lead to a cardiac event, again mainly or only in susceptible patients [7].

#### Pathway 2c

Direct respiratory reflexes, as described for pathway 1c, may lead to dysfunction of the ANS and cardiovascular autonomic function, which can in turn lead to disruption of the cardiac rhythm and (fatal) arrhythmia [1,8,11] in susceptible patients. Disturbances in the control of heart rate in response to respiratory reflexes induced by particulate pollution were originally suggested by two observational studies [57,58], but experimental animal evidence also supports this hypothesis [7].
